# Prediction of the final size for COVID-19 epidemic using machine learning: A case study of Egypt

**DOI:** 10.1016/j.idm.2020.08.008

**Published:** 2020-08-25

**Authors:** Lamiaa A. Amar, Ashraf A. Taha, Marwa Y. Mohamed

**Affiliations:** a Networks and Distributed Systems Department, Informatics Research Institute, City of Scientific Research and Technological Applications, SRTA-CITY, Egypt; b Multimedia and Computer Graphics Department, Informatics Research Institute, City of Scientific Research and Technological Applications, SRTA-CITY, Egypt; c Department of Mathematics and Computer Science, Faculty of Science, Alexandria University, Egypt

**Keywords:** COVID-19, Regression analysis model, Epidemic model

## Abstract

COVID-19 is spreading within the sort of an enormous epidemic for the globe. This epidemic infects a lot of individuals in Egypt. The World Health Organization states that COVID-19 could be spread from one person to another at a very fast speed through contact and respiratory spray. On these days, Egypt and all countries worldwide should rise to an effective step to investigate this disease and eliminate the effects of this epidemic. In this paper displayed, the real database of COVID-19 for Egypt has been analysed from February 15, 2020, to June 15, 2020, and predicted with the number of patients that will be infected with COVID-19, and estimated the epidemic final size. Several regression analysis models have been applied for data analysis of COVID-19 of Egypt. In this study, we’ve been applied seven regression analysis-based models that are exponential polynomial, quadratic, third-degree, fourth-degree, fifth-degree, sixth-degree, and logit growth respectively for the COVID-19 dataset. Thus, the exponential, fourth-degree, fifth-degree, and sixth-degree polynomial regression models are excellent models specially fourth-degree model that will help the government preparing their procedures for one month. In addition, we have applied the well-known logit growth regression model and we obtained the following epidemiological insights: Firstly, the epidemic peak could possibly reach at 22-June 2020 and final time of epidemic at 8-September 2020. Secondly, the final total size for cases 1.6676E+05 cases. The action from government of interevent over a relatively long interval is necessary to minimize the final epidemic size.

## 1 Introduction

Exclusive outbreaks of a novel epidemic Coronavirus (COVID-19) worldwide lead the researchers and scientists in different fields to look for the ways to address the challenges of this virus and work on overcoming the epidemic. At the end of June 2020, more than 10 million infected cases had been reported in 188 regions and territories since the first declaration of December 2019 in Wuhan City, Hubei region, China (Kuniya, 2020). The number of identified cases has been increasing rapidly over the world, so different researches and projects faced new recent challenges to forecast the peak of the epidemic to help the governments make decisions for limiting the spreading of the malady. In Egypt, the number of reported cases has increased daily after the first case was declared on 14 February 2020. In June 2020, exceed 60 thousand infections and about three thousand deaths cases had been reported and daily reports were published by the Health and Population Ministry since the starting date of virus till now. The most important statistics of the current situation to combat the emerging coronavirus in Egypt according to ministry reports compared to the world; First, Egypt ranked 77th the dying toll of the whole number of individuals infected with the virus. The ratio is equivalent to (4.346%) after Lithuania (4.29%) and Guatemala (4.27%). Egypt is preceded by Curacao, America (4.348%). Second, with respect to the recovery rate, the ranking is 184th and the ratio is equivalent to (27%), followed by Eretria (26.1%). Third, with respect to the terms of total injuries per million people, the ranking is 103rd (682 cases/million), compared to all countries and regions worldwide. Finally, the ranking is 23rd with respect to the number of individuals infected with the virus among 215 regions and countries around the globe (Ministry of health and population, 2020).

When the Coronavirus emerged in Wuhan city, Egypt began its preventive procedures against this fatal virus as one of the most attractive tourism countries worldwide. Immediately, isolation departments in hospitals of fever were delegated with dealing with such cases. Health and Population Ministry played an important role in raising awareness and monitoring the global epidemiological situation around the clock. Where the new virus was described and clarified to the people at all private clinics and hospitals. The ministry informed all the citizens’ country wide to immediately report cases and then refers them to the nearest chest or fever hospital. The ports of Alexandria, Red Sea, Damietta, and Port Said declared the emergency to face the Coronavirus in conjunction with the departments of quarantine in each port. In order to keep people and visitors safe from the danger of this virus, the quarantine officials were present to inspect and examine all arrivals during the ports’ reception for boats and ships, particularly those from the countries where the disease was appeared and spread. An operating control room was established in coordination to the quarantine departments to continue checking the arrivals. When a person is suspected to have contacted a patient with coronavirus, he or she will be immediately isolated.

About the successful experiences that took place these days, the Egyptian Health Minister announced the success of an injection experiment for critical cases by plasma from recover patients, increasing the recovery rate of discharge in the hospital increased too. The challenge now is how to estimate the peak of a pandemic keeping in mind all the efforts that has been made in all directions.

## 2 Literature review

The major challenges associated with COVID-19 is delivering several works to overcome the epidemic and take the necessary precautions needed to educate people and support government efforts that have been made to stabilize the country. The challenge now for researchers all over the world is how to estimate the peak of a virus keeping in mind all the efforts in all directions. In this section, we will review some related works in this direction. The authors in (Wagida) use an Epidemic Calculator that uses (SEIR compartmental model) with Health of Egyptian Ministry of and population released regular reports (14 February 2020 to 11 May 2020). For the highest estimated case, mortally rate (7.7 percent), the number of hospitalized people predicted to peak in mid-June, with a total of 20,126 hospitalized cases of 20,126 individuals and total expected deaths 12,303. The author recommends reinforcement of the Egyptian preventive and control measures to get better the case fatality rate (CFR) and the numeral of cases to the least possible as we reach the peak. It is most important that appropriate quarantine measures retained before the end of June 2020.

Machine learning and statistical modeling approaches were used to predict and estimate the ending stage of COVID-19 in Kuwait (especially with time-different infection rates and individual contact numbers) (Almeshal et al., 2020). Results indicate that the estimated number of reproductions in Kuwait is 2.2, with data up to 19 April 2020 and before the repatriation plan. The results indicate that a high contact rate among the population denotes that the epidemic peak value will not reach and the country needs more strict intervention measures. Moreover, the prediction of the peak date and simulation of the variations that could be happening by the social behaviours of Egyptians during Ramadan (the holy month) (El Desouky, 2020). Mainly, the peak will depend on the behaviours of people towards social distancing and hygiene measures. The strategies of lockdown in Egypt have a positive effect on the delay of the epidemic peak, providing more time to help the global health sector to encompass the situation. The Egyptian government should monitor the reported cases daily along with the performance of citizens in the coming month to identify the proper strategies to flatten the curve as much as possible.

Numerical approaches and logistic model are used in (Ahmed et al., 2020) for COVID-19 analysis. Researchers suggest three recognized numerical methods (Euler’s method, Runge–Kutta method of order two (RK2) and of order four (RK4)) for solving such equations about the Global health care and suggest important notes. Numerical results may use to guess the number susceptible to infection, recovered, and quarantined individuals in the future to support the foreign efforts to develop their intervention services and further prevention.

In (Pirouz et al., 2020), processing sustainable development was studied using the classification of confirmed cases of COVID-19. Therefore, using one of the Artificial Intelligence (AI) techniques, the community data handling system (GMDH) type of neural network used binary classification modeling. The proposed model was developed as a case study in China’s Hubei province. Some important parameters, namely maximum, minimum, and average daily temperature, the density of a city, relative humidity, and wind speed, parsed as the input data set, and picked the number of confirmed cases as the output data set for 30 days.

The proposed model of binary classification provides greater capacity for accuracy in predicting the reported cases. Furthermore, regression analysis and the pattern of reported cases relative to the variations of the daily weather parameters (wind, humidity, and average temperature) have been performed. The results showed that relative humidity and the maximum daily temperature had the greatest impact on the actual cases. The relative humidity in the confirmed case study was 77.9% on average, positively affected, and the average daily temperature was 15.4 °C on average, affected negatively the real cases.

In (Ranjan, 2020) compares the COVID-19 data from India against several countries as well as key states in the US with a main outbreak, and it is found that the first reproduction number R0 for India is in the expected range of 1.4–3.9. Meanwhile, the ring of growth of infections in India is very close to that in Washington and California. Exponential and classical models of susceptible-infected-recovered (SIR) depend on current data used to render frequent short-ring and long-term predictions. From the SIR model, it is estimated that India will enter stability by the end of May 2020 with the final size of epidemic near to 13,000. Though, if India enters the group transmission point, the approximation will be invalid. The effect of social distancing is also measured by analyzing data from various geographical locations, once again with the presumption of no group transmission.

Researchers and communities are provided new AI and huge data applications to get better the COVID-19 epidemic situation, and also further studies in stopping COVID-19 outbreak to control the virus situation (Pham & Nguyen, 2020). The paper presented a survey on the state-of the-art solutions in the action against the COVID-19 pandemic. In previous studies, researchers depended on various proposed methods and analysed the results based on some of the parameters and models.

The main contributions of our study are as follows:

1 Using machine learning and the best regression analysis model to predict the rate of spread of COVID-19 for a month in Egypt.
2 Presenting mathematical models to predict the spread of COVID-19 in Egypt estimate the epidemic size and predict an ending phase of the epidemic.

## 3 Methodology

### 3.1 Study area

Egypt is an African country found in the Eastern Mediterranean vicinity in line with the classification of the World Health Organization (WHO) and categorized as a lower-middle-profits country with respect to a World Bank category. The total inhabitance of Egypt is almost 100 million individuals and almost 8% of them are exceeding 60 years old. About 1.7% of the entire inhabitance lives under the national poorness line. Systems of health in Egypt, like African countries, have low resources to confront the pandemic. Egypt features a physician density of 0.79 physicians/1000 individuals and a single bed capacity of 1.6 beds/1000 individuals. The demographic structure of Egypt highlights a particular nature that varies from other European and Asian countries where the middle age of the Egyptians is 24.6 years (the middle age for Chinese is 38.4 years). As they were 4.23% of Egyptian individuals have almost 65 years. The infected countries’ experiences (in Europe and Asia) appeared that elderly individuals over 60 years and individuals who have weakening maladies are most defenceless to genuine grades of COVID-19. In this manner, the Egyptian young may act as a defensive line to constrain the spread of the widespread around the world.

### 3.2 Data sources

**Fig. 1:**
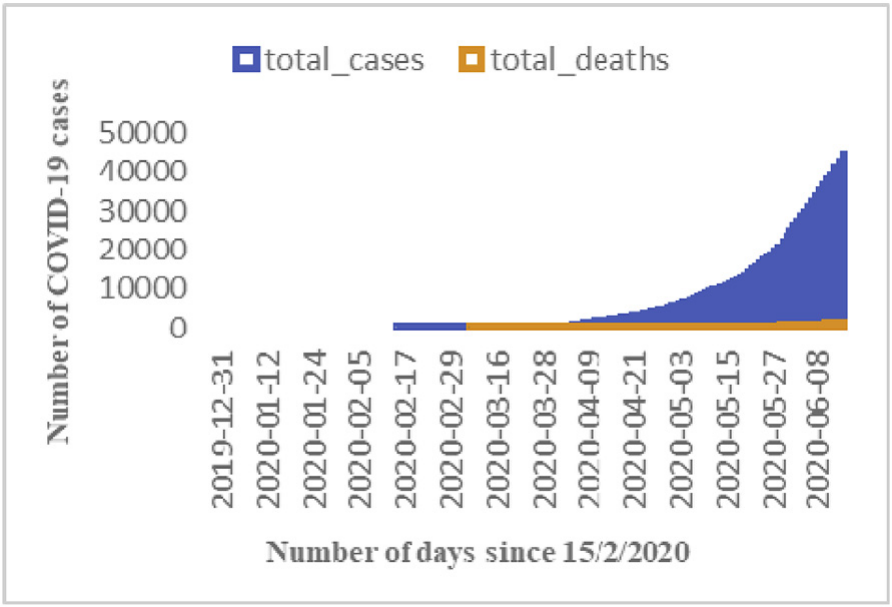
Real data for Cumulative total new confirmed cases and died cases.

Daily, prevalence data of COVID-19 is reported by the Egyptian Ministry of health and population (Ting et al., 2020) and

www.ourworldindata.org/coronavirus-source-data. Fig. 1 presents the COVID-19 confirmed, and mortal cases distribution in Egypt for the period from 15 February to 15 June 2020. It is easy to observe the spread is exponential growth, which needs to be controlled. Its future epidemiological progression is still ambiguous as it spreads randomly.

### 3.3 Regression models

Regression model analysis is a subset of Machine Learning (ML) algorithms (Singh & Dhar, 2018). A variety of regression models is available including linear and non-linear forms, namely Multiple Linear Regression. Some of these models follow the parametric or the non-parametric approaches for statistical inference. The regression analysis technique is a kind of modeling technique used in epidemiologic research to estimate relationships among sets of variables. ‘‘The regression analysis techniques are a set of ML methods that allow us to forecast continuous results variable (Y) based on one or multiple predictor variables (X). It assumes a linear relationship among the results and the predictor variables’’. Numbers of regression analysis technique s have been applied to forecast the accumulated confirmed COVID-19 within (15 days), the final size of epidemic cases, and the final time of epidemic in Egypt. In this proposed, we consider the following models:

#### 3.3.1 Exponential regression model

It is used to epidemic model cases in which starts growth slowly and then accelerates speedily without bound, or where decrease begins speedily and then the speed reduce to get closer until reach to zero. The equation that describes this model is:

(1) $$\text{y}={\text{a}}_{1}\;e^{a_{2}X}$$

where ɑ_1_ and ɑ_2_ are called the parameters of regression analysis.

#### 3.3.2 Polynomial regression model

A polynomial term turns a linear regression model into a curve but it still qualifies as a linear model. The polynomial models quadratic, third-degree, fourth-degree, fifth-degree, and sixth-degree were used in those situations. The nth order polynomial model in one variable is given by the equation:

(2) $$y=a_{1}.X+a_{2}.X^{2}+a_{3}.X^{3}+a_{3}.X^{4}+...+a_{n}.X^{n}+\varepsilon$$

where (n = 2, ….,6) represents the degree of the models.

The coefficients ɑ_1_, ɑ_2_ … ɑ _n_a1,a2, …,an are called the parameters of regression analysis.

#### 3.3.3 Logit growth regression model

The logit model or (logistic model) is a technique borrowed by machine learning from the field of statistics. The logit model is a regression model that is widely used in epidemiology mathematical models to estimate the growth rate of the epidemic (Batista, 2020). The model assumes an exponential growth at the beginning of the epidemic, followed by a steady increase and finally ending with a declining growth rate. The logit model is presented by equation (3) as:

(3) $$\frac{1}{C}\frac{dI}{dt}=C_{r}\left(1-\frac{C}{K}\right)$$

The natural growth equation:

(4) C=KeCrtCrAt+A

where A = K– C0C0A = K–C0C0 and t = 0 = t = , assuming A, k > C_0_ K > C0.

Hence, if *C* is an accumulated number of cases, *C*_*r*_ defined as the rate of infection cases, K is the final epidemic size, t is the time, dCdt is the growth rate reaches its maximum when dC2dt2 = 0dC2dt2 = 0 dC2dt2 = 0.

To fit the maximum number of confirmed cases (peak number of cases) of the infected population C_*Peak*_ and coefficient, t_Peak_ and $\frac{\text{dC}}{{\text{dt}}_{\text{Peak}}}$ are defined by the formulas.

(5) $${\text{C}}_{\text{Peak}}=\frac{\text{K}}{2}$$

(6) $${\text{t}}_{\text{Peak}}=\;\frac{\text{lnA}}{{\text{C}}_{\text{f}}}$$

(7) $$\frac{\text{dC}}{{\text{dt}}_{\text{Peak}}}=\frac{{\text{C}}_{\text{r}}\text{ K}}{4}$$

If *C*_*1*_*, C*_*2*_ *… C*_*f*_ represent the number of cases at times *t*_*1*_*, t*_*2*_*, …, t*_*final*_ then the final size predictions of the epidemic based on these data are *K*_*1*_*, K*_*2*_*, …, K*_*f*_ the predicted final epidemic size is presented by equation (8) by iterated Shanks transformation (Bender & Orszag, 1999).

(8) $$\text{K}=\frac{{\text{K}}_{\text{f}+1}{\text{ k}}_{\text{f}-1}-{\text{ K}}_{\text{f}}^{2}}{{\text{K}}_{\text{f}+1}-2{\text{K}}_{\text{f}}-{\text{K}}_{\text{f}-1}}$$

The logit model presented in equation (4) contains three coefficients: *K, C*_*r*_*, and A* which should be determined by regression analysis because of the nonlinearity of the model.

#### 3.3.4 Regression analysis

• Correlation coefﬁcients

The Correlation coefﬁcient means the force of a linear relationship between two variables. According to Karl Pearson, the coefﬁcient of correlation is a measure or degree of the linear relationship between two random variables X and Y. The values range between −1.0 and 1.0.

The correlation coefficient is denoted by “r”. To find *r* is calculated the Pearson product-correlation with the formula as:

(9) $$\text{r}=\frac{\text{n }\left(\sum \text{XY}\right)-\;\left(\sum \text{X}\right)\left(\sum \text{Y}\right)\;}{\sqrt{\left[\text{n}\sum {\text{X}}^{2}-\;{\left(\sum \text{X}\right)}^{2}\right]\left[\text{n}\sum {\text{Y}}^{2}-\;{\left(\sum \text{Y}\right)}^{2}\right]}}$$

Here, when calculating the correlation coefficient γr between the date and number of real cases in Egypt. There are some predictions that are given as:

o r = 0r = 0, where, there is no correlation between input and output variables.
o r = 1r = 1, there is a strong positive relationship between input and output variables (means if the input variable increases the output variable increase and vice versa).
o r = -1r = −1, there is an inverse relationship between input and output variables (means if the input variable increases the output variable decrease and vice versa).

• Residuals.

Residuals are the measure of the quality of fit straight lines of the suggested models. It is the difference between the observed values of the response variable (YY) and the value of the proposed model. The following formula is used to calculate the residuals:

$$\text{Residuals}={\text{Y}}_{\text{observed}}-{\text{Y}}_{\;\text{models values}}\frac{\text{dC}}{{\text{dt}}_{\text{Peak}}}$$

• Adjusted**-R**^**2**^

In this suggested study, we have calculated both simple and adjusted R^2^ to know which the extra terms n and d terms get pitter the predictive power of proposed methods. Adjusted R^2^ for polynomial regression is defined as the following formula:

$${\text{R}}_{\text{adjusted}}^{2}=1-\frac{\left({\text{SS}}_{\text{residual}}\right)}{\left({\text{SS}}_{\text{total}}\right)}\text{∗ }\left(\frac{\text{n}-1}{\text{n}-\text{d}-1}\right)$$

where n is the number of observations in training datasets and dd is the degree of polynomials in regression models. SS_residual_ SSresidualrepresents the sum of the squared residuals from the regression and SS_total_ represents the sum of the squared difference from the mean of the dependent variables.

## 4 Results and discussion

In this proposed study, we have taken a real dataset for the COVID-19 after the outbreak of the epidemic in Egypt. The ﬁrst case of the COVID 2019 epidemic was found in Egypt on 15 February 2020 after that, things escalated in March, several cases were reported all over the country at the end of March caused of loss of human lives. Although the Prime Minister issued in the 4th quarter of March a package of prudential decisions, there was a closure of all shops and establishments that provide entertainment or recreation, as well as the suspension of studies because of the number of students in schools and universities, is approximately 25 million. However, the COVID-19 epidemic in Egypt is growing in exponential form from 15 February 2020 to 15 June 2020.

The discussed machine learning approaches output the possible number of cases for the next 15 days across the world. In this study, illustrates the predicted trend of the COVID-19 using different regression approaches were utilized to fit the confirmed cumulative cases in Egypt from the start of the outbreak on 15 February 2020 until 15 June 2020 and predict short term forecast to help the government for prevention measures in Egypt. We have been utilized seven regression analysis models namely exponential, quadratic, third degree, fourth degree, ﬁfth degree, sixth degree, exponential polynomial, and logit respectively for the COVID-19 dataset. Machine learning approaches are implemented using the python library.

First of all, the correlation coefﬁcient calculate between the date and number of conﬁrmed cases of COVID-19 spread up of Egypt from 15th February to 15th June 2020 to test the correlation between them. The correlation coefficient is γ = 0.8435r = 0.8435, which is very close to 1, indicating that there is a strong statistical correlation between the two variables, date and the number of conﬁrmed cases spread of COVID 2019.

### 4.1 Regression models

The regression models’ approach for epidemic analysis are trained and after that tested on real data using the date and the number of confirmed cases as the label for the corresponding day presented in the above Table 1. Egypt datasets were separated into training datasets from 15-February-2020 to 31-May-2020 and testing dataset from 1-June to 15-June 2020.

**Table 1:** training dataset of COVID 19 of Egypt from 15 February 2020 to 31-May-2020 and testing dataset from 1 June-2020 to 15 June-2020.

Date	Number of new cases	Accumulated cases	Date	Number of new cases	Accumulated cases
15-Feb	1	1	16-Apr	155	2505
16-Feb	0	1	17-Apr	168	2673
17-Feb	0	1	18-Apr	171	2844
18-Feb	0	1	19-Apr	188	3032
19-Feb	0	1	20-Apr	112	3144
20-Feb	0	1	21-Apr	189	3333
21-Feb	0	1	22-Apr	157	3490
22-Feb	0	1	23-Apr	0	3490
23-Feb	0	1	24-Apr	169	3659
24-Feb	0	1	25-Apr	433	4092
25-Feb	0	1	26-Apr	227	4319
26-Feb	0	1	27-Apr	0	4319
27-Feb	0	1	28-Apr	463	4782
28-Feb	0	1	29-Apr	260	5042
29-Feb	0	1	30-Apr	226	5268
01-Mar	0	1	01-May	269	5537
02-Mar	1	2	02-May	358	5895
03-Mar	1	3	03-May	298	6193
04-Mar	0	3	04-May	272	6465
05-Mar	0	3	05-May	348	6813
06-Mar	0	3	06-May	388	7201
07-Mar	0	3	07-May	387	7588
08-Mar	12	15	08-May	393	7981
09-Mar	34	49	09-May	495	8476
10-Mar	6	55	10-May	488	8964
11-Mar	4	59	11-May	436	9400
12-Mar	1	60	12-May	346	9746
13-Mar	20	80	13-May	347	10093
14-Mar	13	93	14-May	338	10431
15-Mar	0	93	15-May	398	10829
16-Mar	17	110	16-May	399	11228
17-Mar	16	126	17-May	491	11719
18-Mar	40	166	18-May	510	12229
19-Mar	30	196	19-May	535	12764
20-Mar	14	210	20-May	720	13484
21-Mar	46	256	21-May	745	14229
22-Mar	29	285	22-May	774	15003
23-Mar	9	294	23-May	783	15786
24-Mar	33	366	24-May	827	16613
25-Mar	39	442	25-May	652	17265
26-Mar	76	456	26-May	702	17967
27-Mar	14	495	27-May	789	18756
28-Mar	39	536	28-May	910	19666
29-Mar	41	576	29-May	1127	20793
30-Mar	40	609	30-May	1289	22082
31-Mar	33	656	31-May	1367	23449
01-Apr	47	710	01-Jun	1536	24985
02-Apr	54	779	02-Jun	1399	26384
03-Apr	69	779	03-Jun	1152	27536
04-Apr	0	985	04-Jun	1079	28615
05-Apr	206	1070	05-Jun	1152	29767
06-Apr	85	1073	06-Jun	1348	31115
07-Apr	3	1322	07-Jun	1497	32612
08-Apr	249	1560	08-Jun	1467	34079
09-Apr	238	1699	09-Jun	1365	35444
10-Apr	139	1794	10-Jun	1385	36829
11-Apr	95	1939	11-Jun	1455	38284
12-Apr	145	2065	12-Jun	1442	39726
13-Apr	126	2190	13-Jun	1578	41304
14-Apr	125	2350	14-Jun	1667	42980
15-Apr	160		15-Jun	1618	44598

In this regard, we have used exponential, quadratic, third-degree, fourth-degree, fifth-degree, and sixth-degree polynomial regression models. In these proposed regression models, we have used independent variable X and dependent variable Y. From training dataset was calculated the coefficient for equation (1&2) for all regression models as shown in Table 2 and represent them in the equations below as the following:

$$Y=20.7\;e^{0.047X}$$

$$Y=3.0618\;X^{2}-338.34607\;X+6282.77$$

$$Y=0.0376\;X^{3}\;-6.310\;X^{2}+291.506\;X\;-2924.12$$

$$Y=0.0003\;X^{4}-0.076\;X^{3}+5.887\;X^{2}+-164.741\;X\;+1092.87$$

$$Y=2.705\text{e}-06\;X^{5}-0.0008\;X^{4}+0.0898\;X^{3}-4.558\;X^{2}+89.329\;X\;-421.90$$

$$Y=1.64\text{e}-08\;X^{6}-5.44\text{e}-06X^{5}+0.0008\;X^{4}-0.047\;X^{3}+1.170X^{2}-8.533X+0.559$$

**Table 2:** The coefficient parameters for the regression models.

Model	α_1_	α_2_	α_3_	α_4_	α_5_	α_6_	Ε
Exponential	20.7	0.047	–	–	–	–	–
Quadratic	−338.346	3.0618	–	–	–	–	6282.77
Third	291.506	−6.310	0.0376	–	–	–	−2924.12
Fourth	−164.741	5.887	−0.076	0.0003	–	–	1092.87
Fifth	89.329	−4.558	0.0898	−0.0008	2.705e-06	–	−421.90
Sixth	−8.533	1.170	−0.047	0.0008	−5.44e-06	1.64e-08	0.559

**Fig. 2:**
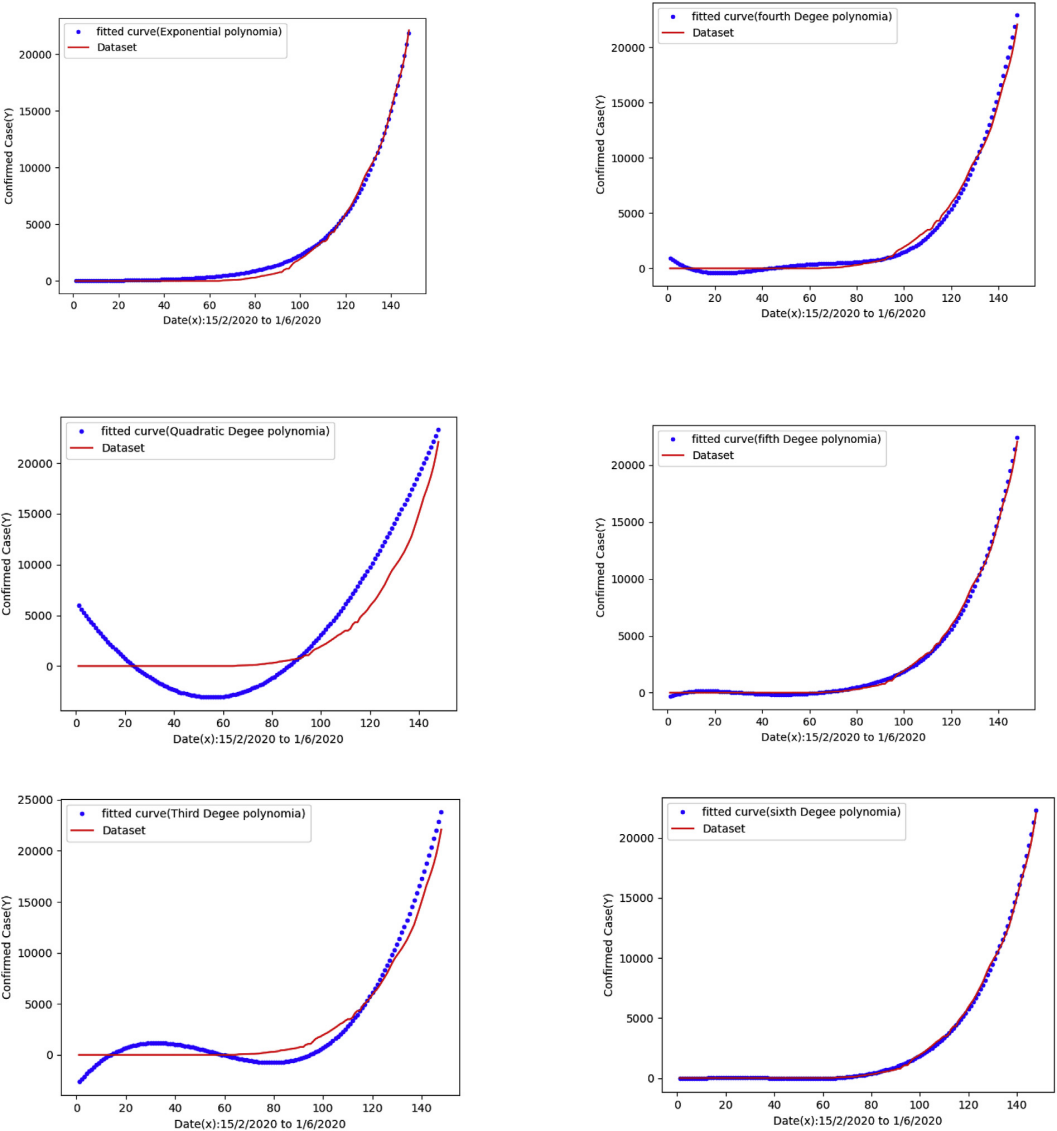

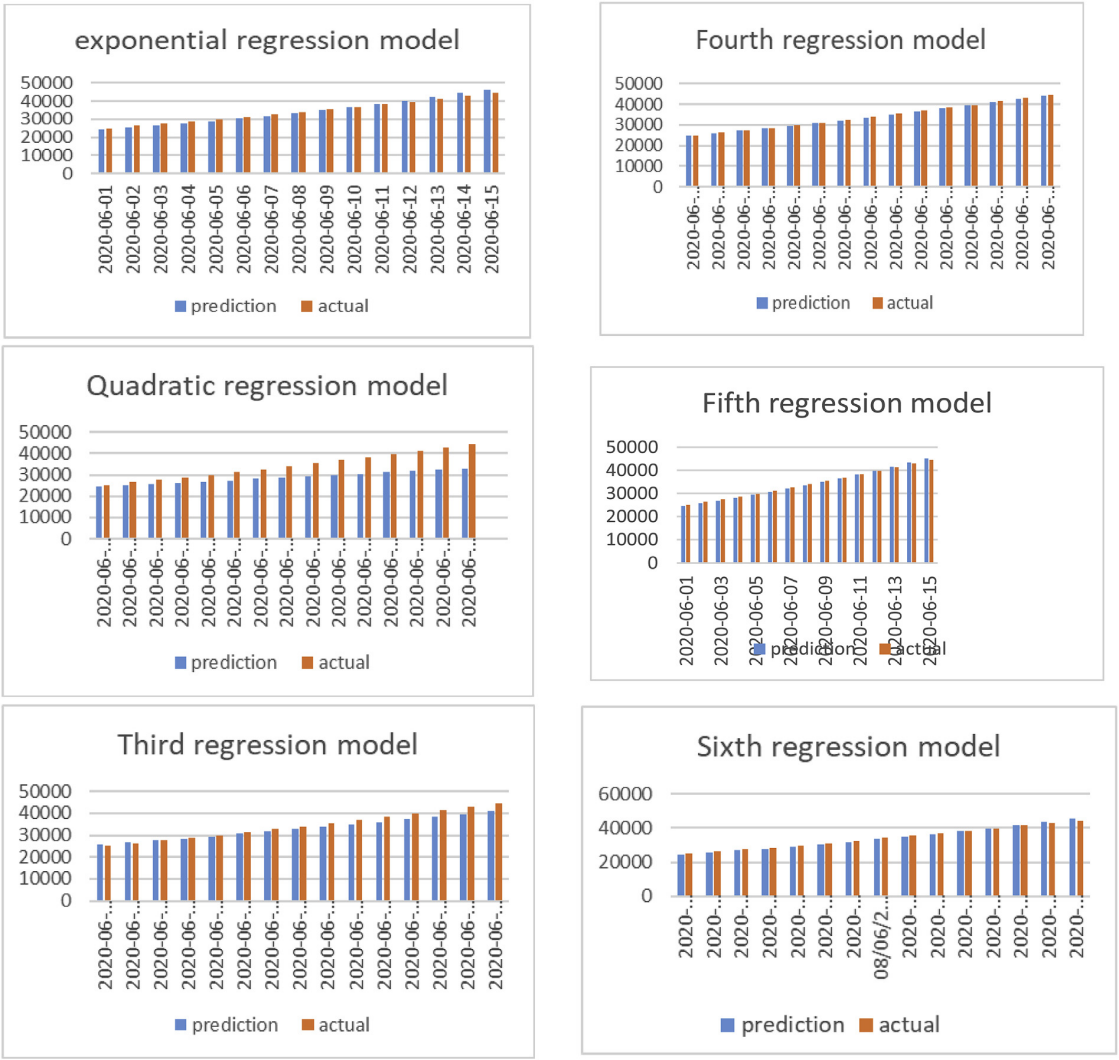
(a) Fitted curves with training data based on regression (b) Comparison of the real case and the predicted models: exponential, quadratic, third degree, fourth-degree, results of the proposed models: exponential, quadratic, third- Fifth-degree and sixth-degree polynomial degree, fourth-degree, fifth-degree and, sixth-degree. Polynomial on the testing dataset of Egypt COVID-19.

We show below in Fig. 2a the results of actual cases of the proposed fitted regression analysis-based models namely: exponential, quadratic, third-degree, fourth-degree, fifth-degree, sixth-degree polynomial, and logit growth for the training datasets of the COVID-19 in Egypt from (12-Feb to 31-May). As shown in Fig. 2b a comparison of the actual results and predicted results of the proposed models: exponential, quadratic, third degree, fourth degree, fifth degree, sixth degree polynomial and, logit growth for the testing dataset of the COVID-19 from (1-Jun to 15-Jun). We observed from the figures that the result of the proposed fourth-degree, fifth-degree, and sixth-degree polynomial methods are very close to actual results.

**Fig. 3:**
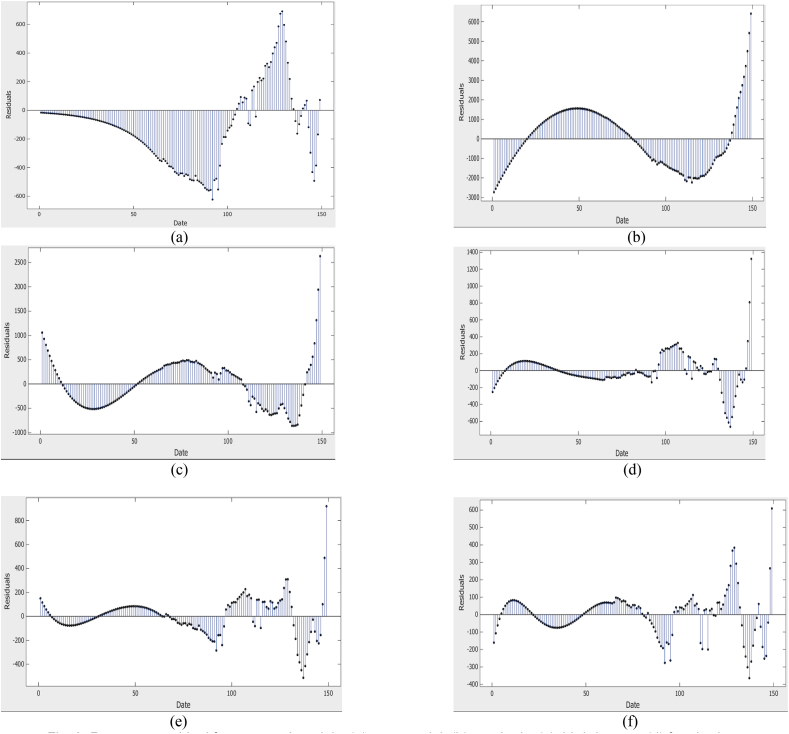
Represent residual for proposed models: (a) exponential, (b).quadratic, (c) third-degree, (d) fourth-degree, (e) fifth-degree and (f) sixth-degree polynomial for dataset Egypt COVID-19

In regression analysis, residuals play an important role in the COVID-19 outbreak data analysis in Egypt. All the residuals for the proposed methods exponential, quadratic, third-degree, fourth-degree, fifth-degree and, sixth-degree polynomials are calculated and plotted as in Fig. 3. We observed that the exponential, fourth-degree, fifth-degree, and sixth-degree polynomial regression models give strong patterns.

**Fig. 4:**
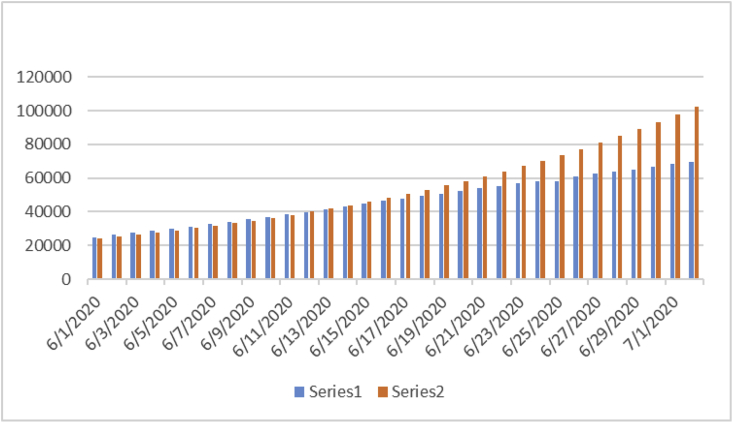
The predicted final size of epidemic in Egypt after one month.

Finally, Figs. 2 and 3 show that the better-fitted results and residuals were the exponential, fourth-degree, fifth-degree and, sixth-degree polynomial, respectively. Therefore, the proposed models: exponential, fourth-degree, fifth-degree, sixth-degree gave excellent results to predict the next 15 days. The fourth-degree regression model has given excellent result to predict the next 1 month as shown in Fig. 4, so it is very useful for future prediction of the COVID-19 outbreak in Egypt for one month so, the government will take a good decision.

### 4.2 Logit growth regression

**Fig. 5:**
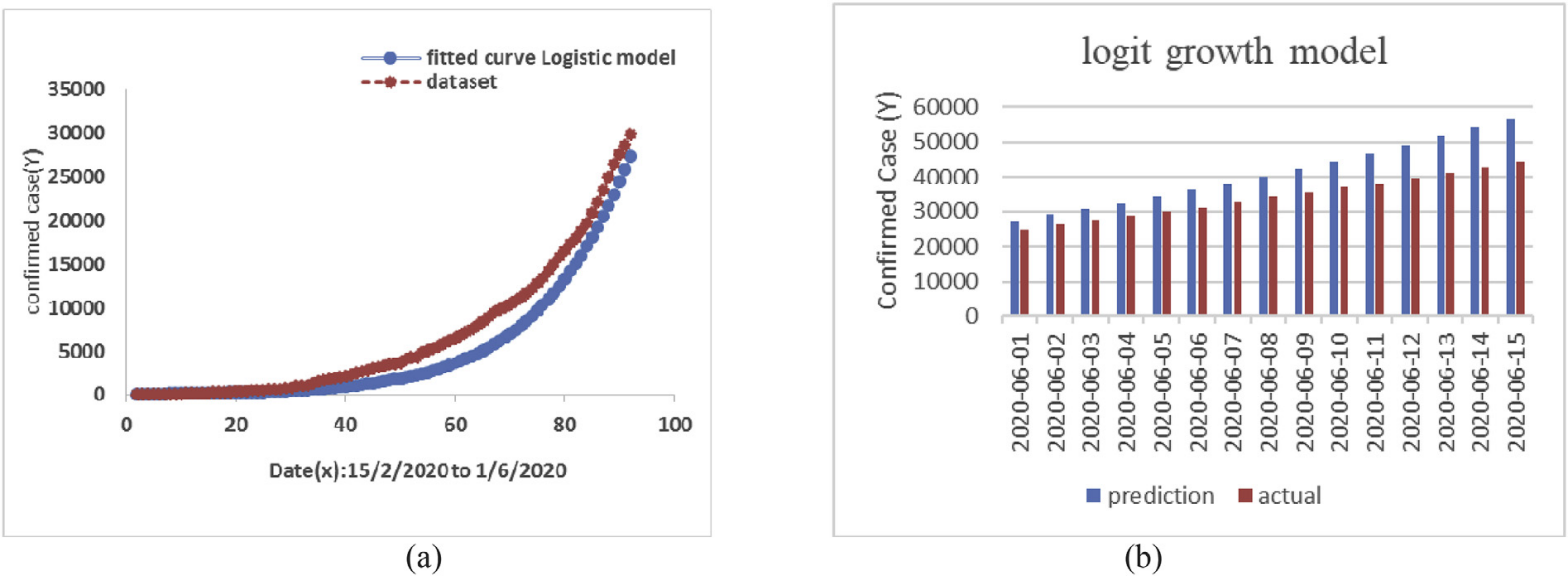
(a).Fitted curves with training data based on logit Fig. 5. (b) Comparison of the real case and the predicted growth regression mode. Results of the logit model applied on the testing dataset of Egypt COVID19.

We utilized the logit growth regression approach to fit the confirmed cumulative cases in Egypt from the start of the outbreak on 15 February 2020 until 15 June 2020 and represent on the training dataset and compared the prediction with the testing data as shown in Fig. 5.

**Fig. 6:**
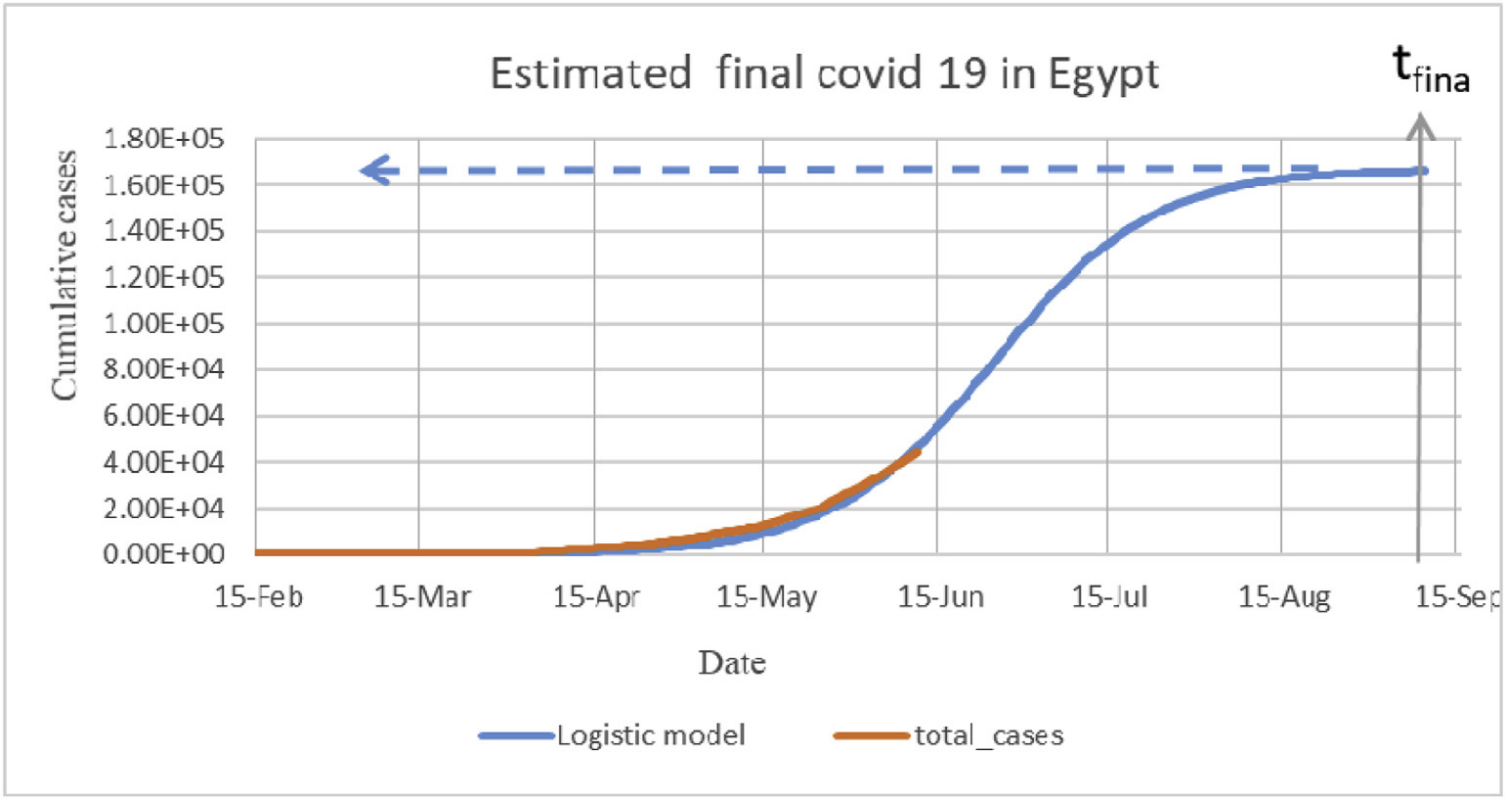
The predicted final of coronavirus in Egypt for epidemic (data until 15 Jun 2020).

**Fig. 7:**
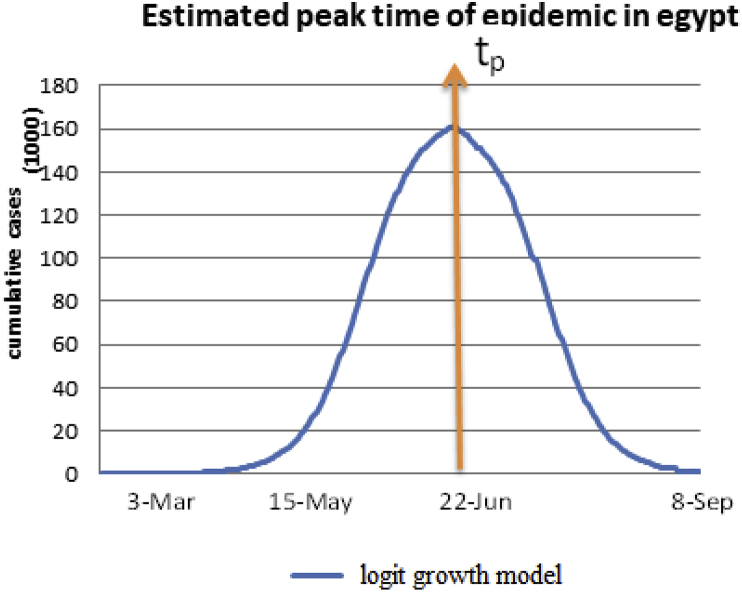
Illustrated the phases of coronavirus epidemic in Egypt the first phase at 3-Mar, second phase at 15-May and third phase (peak) at 22-Jun and final epidemic at 8-Sep.

From the below Fig. 6, we show that the estimated final of the epidemic t_final_ was probably on 8 Sep 2020. The Shanks a transformation equation was used for the predicted of the final epidemic size K. It appears that the prediction of the logit model reaches to the final size almost at 1.6676E 05 cases (see Fig. 7).

Table 3 represents the coefficients A, K, and C_r_ of equation (4) and the phases of the epidemic time that were estimated by all regression analysis models.

**Table 3:** Estimated Logit model coefficients.

Parameters	Value
A	410.8346
K	1.6676 E +05
C_r_	0.537
Estimated start phase of epidemic	15 May 2020
Estimate the peak date of epidemic	22 Jun 2020
Estimate the final time of epidemic Root mean square error	8 Sep 2020 350.3935

Notes: Coronavirus affected by phases as shown in Fig. 6

1 The first phase: start case infection and slow growth of the epidemic. $\;t\;<\;t_{p}-2/c_{r}$
2 Second phase: fast growth infection. $t_{p}-\frac{2}{c_{r}t}<t<t_{p}$
3 Third phase: steady-state and slow growth (peak). $t\approx t_{p}$
4 Fourth phase: start decrease. $t>2\;t_{p}$

The simulation was carried out the parameters estimated namely: start phase of the epidemic, the peak date of epidemic, the start of ending phase of the epidemic and the root mean square error.

Finally the measure metrics for different of regression models was shown in Table 4 the calculated results of the Sum of Square regression (SSR), residual square (R2)R^2^) and, adjusted-(R^2^) for all proposed models, which highlights the best fitting of the suggested models.

**Table 4:** the Coefficient parameters for the regression models.

*Model*	SSR	R^2^	Adjusted-R^2^
Exponential	15,941,459.4	0.997	0.997
Quadratic	1,184,486,998	0.919	0.918
Third	146,622,229.9	0.980	0.980
Fourth	31,056,054.7	0.995	0.995
Fifth	7,957,225.5	0.999	0.999
Sixth Logit	4,610,517.3 3,503,935	0.999 0.999	0.999 0.999

## 5 Conclusion

A forecast of COVID-19 spread in Egypt was carried out using various statistics and machine learning modeling approaches. The forecast was based on the data from 15 February 2020 until 15 June 2020. These models also predicted the outbreak of the COVID-19 in Egypt for the next 15 days, one month, the final size of the infected cases, and the final time of the epidemic. Here, we have found out that the best of the proposed models namely exponential, fourth-degree, the fifth-degree, and sixth-degree polynomial are strong residual and prediction for the next 15 days and also the fourth-degree model has given an excellent prediction for one month. These models are very useful for the Egyptian government for managing the COVID-19 outbreak for the next months. The study aimed to investigate and assess the effectiveness of preventive measures of the government of Egypt to control the spread of COVID-19. In this study, by applying the logit growth regression model to the daily reported cases of COVID-19, we have estimated that the peak epidemic in 22-June 2020 could possibly reach the final time in 8-September 2020. Of course, this type of peak forecasting would contain the essential uncertainty due to the possibility of some big changes in the social and natural (climate) situations. Moreover, our result suggests that the epidemic of COVID-19 in Egypt would not end so quickly.
